# IL-1β and TNF-α Modulation of Proliferated and Committed Myoblasts: IL-6 and COX-2-Derived Prostaglandins as Key Actors in the Mechanisms Involved

**DOI:** 10.3390/cells9092005

**Published:** 2020-09-01

**Authors:** Angela M. Alvarez, Carlos DeOcesano-Pereira, Catarina Teixeira, Vanessa Moreira

**Affiliations:** 1 Centre of Excellence in New Target Discovery (CENTD), Butantan Institute, São Paulo, SP 05503-900, Brazil; angela.alvarez@butantan.gov.br (A.M.A.); carlos.ocesano@butantan.gov.br (C.D.-P.); 2 Pharmacology Department, Escola Paulista de Medicina, Universidade Federal de São Paulo, São Paulo, SP 04044-020, Brazil; 3 Pharmacology Laboratory, Butantan Institute, São Paulo, SP 05503-900, Brazil

**Keywords:** myoblast, inflammation, cytokines, proliferation, myogenesis, muscle regeneration

## Abstract

In this study, we investigated the effects and mechanisms of the pro-inflammatory cytokines IL-1β and TNF-α on the proliferation and commitment phases of myoblast differentiation. C2C12 mouse myoblast cells were cultured to reach a proliferated or committed status and were incubated with these cytokines for the evaluation of cell proliferation, cyclooxygenase 2 (COX-2) expression, release of prostaglandins (PGs) and myokines, and activation of myogenic regulatory factors (MRFs). We found that inhibition of the IL-6 receptor reduced IL-1β- and TNF-α-induced cell proliferation, and that the IL-1β effect also involved COX-2-derived PGs. Both cytokines modulated the release of the myokines myostatin, irisin, osteonectin, and IL-15. TNF-α and IL-6 reduced the activity of Pax7 in proliferated cells and reduced MyoD and myogenin activity at both proliferative and commitment stages. Otherwise, IL-1β increased myogenin activity only in committed cells. Our data reveal a key role of IL-6 and COX-2-derived PGs in IL-1β and TNF-α-induced myoblast proliferation and support the link between TNF-α and IL-6 and the activation of MRFs. We concluded that IL-1β and TNF-α induce similar effects at the initial stages of muscle regeneration but found critical differences between their effects with the progression of the process, bringing new insights into inflammatory signalling in skeletal muscle regeneration.

## 1. Introduction

Skeletal muscle is a dynamic tissue with the ability to regenerate successfully following injury and to adapt in response to growth or exercise. Skeletal muscle stem cells, also named satellite cells, are the main drivers of the plasticity, maintenance, and regeneration of skeletal muscle [1,2,3]. Under resting conditions, these cells are mitotically and physiologically quiescent, but in response to increased physiological muscle loading or injury, they are activated and re-enter the cell cycle to generate satellite cell-derived myoblasts. Myoblasts subsequently proliferate, migrate, differentiate, and fuse to form multinucleated muscle fibres to repair damaged myofibrils or form new myofibrils [4,5]. The process of muscle regeneration, from satellite cell activation to fibre repair, is finely regulated by transcription factors, such as Pax7 and Pax3, and by myogenic regulatory factors including MyoD, Myf5, Myf6, and myogenin, which are expressed in a coordinated fashion and modulated by diverse inflammatory mediators, such as cytokines and prostaglandins [5,6].

The regeneration process after injury involves the systematic activation of innate immune responses. The early inflammatory cascade involves mainly pro-inflammatory macrophages (M1 type) that secrete several pro-inflammatory cytokines, including interleukin-1β (IL-1β), tumour necrosis factor-α (TNF-α), and interleukin-6 (IL-6), which are recognised as key mediators of the initial inflammatory response in the process of skeletal muscle regeneration. IL-1β has been reported to be a pivotal mediator, especially early in the regeneration process, in damaged muscle tissues, acting (i) by amplifying chemoattractants and recruiting immune cells to the site of injury and (ii) by directly influencing the intrinsic capabilities of myoblasts [7,8]. Indeed, following skeletal muscle injury, the expression of IL-1β is highly upregulated in muscle cells and in neutrophils and macrophages recruited around the injured area [7]. Moreover, IL-1β has been shown to increase the proliferation of primary skeletal muscle satellite cells and C2C12 cells in culture [9] and to increase nuclear factor kappa B (NF-κB) activity [10]. A series of in vitro experiments using satellite cells from IL-1α/β double-knockout (IL-1KO) mice revealed that IL-1KO myoblasts have impairments in terms of both proliferation and differentiation that were reversed by exogenous IL-1β administration in culture [11]. Despite the evidence of the important roles played by IL-1β in skeletal muscle cell regeneration, the mechanisms involved in the effects of IL-1β remain poorly understood.

TNF-α levels in injured muscle have been shown to rise dramatically due to a strong increase in its synthesis by injured myofibrils and its release by infiltrating inflammatory cells [12,13,14,15]. While a transient increase in intramuscular TNF-α levels after injury is required for muscle regeneration and repair, a persistent high concentration of this mediator inhibits myogenesis and limits recovery, leading to muscle wasting [8,16,17]. Increased synthesis of TNF-α, observed in the early phase of differentiation of C2C12 myoblasts in culture, is positively associated with regeneration, since its inhibition prevents the process of muscle repair [16,18]. In parallel to TNF-α release, an increased expression of TNF-α receptors is found in injured muscle fibres [13,14], which has been shown to regulate the exiting of the cell cycle and the initiation of myogenic differentiation [19]. TNF-α at low concentrations has been reported to induce the proliferation and differentiation of satellite cells by activating p38 MAPK signalling [20,21,22], thus promoting muscle repair. However, the mechanisms involved in the TNF-α regulation of distinct steps of muscle regeneration remain to be clarified.

IL-6, a cytokine produced by inflammatory cells, satellite cells, and myofibers, is reported to increase the proliferation of satellite cells and the regeneration of damaged myofibers at low levels. In contrast, increased levels of this cytokine at chronic stages of inflammation are related to skeletal muscle wasting [23,24]. Both IL-1β and TNF-α have been demonstrated to induce the release of IL-6 by myoblasts [9,10] and myotubes [25] in culture. However, the role of IL-6 in the effects triggered by TNF-α and IL-1β in skeletal muscle regeneration is unclear. IL-6 participation in TNF-α- induced reduction of MyoD expression in myoblasts has been suggested [26]. Thus, while the relevance of these cytokines to the regeneration process during exercise and regeneration is well demonstrated, the precise roles of these inflammatory mediators in the distinct steps of the process and the biological significance of their crosstalk with IL-6 remain to be clarified.

Skeletal muscle produces specific cytokines known as myokines, such as myostatin, irisin, osteonectin, and interleukin 15 (IL-15), which are released in response to vigorous and prolonged exercise and promote the regeneration of damaged myofibrils [23,27,28]. Several novel myokines have also been reported, and currently, skeletal muscle is considered a secretory organ because myokines have been described to exert autocrine and paracrine functions [29,30,31]. Clearly, these mediators have a substantial impact on muscle growth and function, and these properties raise questions about the participation of myokines in IL-1β and TNF-α effects on proliferation.

In addition to synthesising cytokines and myokines, myoblasts produce prostaglandins (PGs), which are a class of lipid mediators involved in skeletal muscle regeneration. Several in vivo and in vitro evidences indicate that cyclooxygenase (COX)-derived PGs regulate the activation, proliferation, migration, and fusion of myoblasts [24,32,33,34,35]. During muscle regeneration, the main effect of prostaglandin E_2_ (PGE_2_) is the satellite cell proliferation, which is an effect mediated by the EP4 receptor; losses of the PGE_2_ signaling lead to impaired muscle regeneration [35,36]. Muscle injury in mice leads to PGE_2_, PGD_2_, PGI_2_, and PGF_2α_ production in activated myoblasts, and IL-1β and TNF-α were reported to stimulate PGE_2_ release in rat skeletal muscle [24,37,38,39]. However, the participation of these lipid mediators in proliferation induced by the cytokines is still unclear.

On these bases, with the aim of understanding the role of the pro-inflammatory cytokines IL-1β and TNF-α in skeletal muscle regeneration, we compared their effects on the proliferation and commitment phases of myoblast differentiation, focusing on (i) the participation of PGs and IL-6, (ii) the release of myokines, and (iii) activation of the myogenic regulatory factors Pax7, Myf5, MyoD, and myogenin. In this study, we show for the first time that IL-1β and TNF-α have positive proliferative effects through IL-6 via engagement of the IL-6 receptor α (IL-6Rα). We also demonstrate that the effect of IL-1β involves cyclooxygenase 2 (COX-2)-dependent PGs. These cytokines induce distinct effects on the activation of myogenic factors at the proliferative and commitment stages. This study brings new insights into molecular mechanisms triggered by IL-1β and TNF-α in skeletal muscle regeneration.

## 2. Materials and Methods

### 2.1. C2C12 Culture and Pro-Inflammatory Stimulus

Mouse skeletal muscle cell line C2C12 (American Type Culture Collection, Manassas, VA, USA) was grown to confluence in Dulbecco’s Modified Eagle’s Medium (DMEM) (Gibco, Grand Island, NY, USA) supplemented with 10% heat-inactivated foetal bovine serum, 1% L-glutamine (Gibco), 1% penicillin, and 1% streptomycin, at conditions of 37 °C and 5% CO_2_. Then, cells were seeded in 96-well or 6-well plates (Corning, Corning, NY, USA) coated with 2% gelatine (Sigma-Aldrich, Saint Louis, MO, USA) and (i) kept in culture for 48 h for studies in the proliferative stage or (ii) washed with phosphate buffer saline (PBS) 1× on day 5 and then cultured in differentiation medium (DMEM supplemented with 2% horse serum, Sigma-Aldrich) for 72 h for studies in commitment stage. At these time points, cytokines IL-1β and TNF-α (BioLegend, San Diego, CA, USA) at 10 ng/mL were added. Treatments of C2C12 cells with these cytokines in the proliferation and commitment stages had no effect on cell viability (results not shown).

### 2.2. Assessment of Cell Proliferation by BrdU Incorporation

Myoblasts at 0.1 × 10^4^ cells per well were seeded in 96-well clear plates and kept in culture for 48 h. Then, 10 ng/mL of IL-1β or TNF-α was added in DMEM-10% followed by 20 h of incubation. Prior to completing the incubation, 5-bromo-2′-deoxyuridine (BrdU) (1:1000) from the BrdU Cell Proliferation Assay Kit (Cell Signaling Technology, Danvers, MA, USA) was added for 4 h. Following the manufacturer’s instructions, cells were fixed and denatured for 30 min, then washed, and the primary antibody (1:250) was added for 1 h. Cells were washed again and incubated with a secondary antibody conjugated with horseradish peroxidase (HRP; 1:250) for 30 min. Finally, 3,3′,5,5′-tetramethylbenzidine substrate was added for 30 min, the stop solution was added, and the plate was read at 450 nm in a spectrophotometer (SpectraMax from Molecular Devices, San Jose, CA, USA). Results are shown as %BrdU incorporation relative to control (untreated cells).

#### Treatment with Inhibitors

To block the IL-6 receptor, the mouse IL-6Rα antibody (R&D Systems, Minneapolis, MN, USA) was added at 1 μg/mL in culture medium 2 h before the addition of the cytokines. To inhibit COX activity, lumiracoxib (SelleckChem, Houston, TX, USA), a selective pharmacological inhibitor of COX-2, was added at 100 μM in its vehicle (0.1% Tween-80) 6 h after the addition of cytokines.

### 2.3. Measurements of Prostaglandin Release by ELISA

Cells at 2 × 10^4^ cells per well were seeded in 6-well plates and kept in culture for 48 h. At that time, 10 ng/mL of IL-1β or TNF-α was added in the starving media of 1% insulin-transferrin-sodium (ITS, Sigma-Aldrich). After 44 h, cell-free supernatants were collected and kept at −80 °C until analysed. The concentration of prostaglandins was determined by enzyme-linked immunosorbent assay (ELISA) kits for PGE_2_, PGD_2_ (both from Cayman Chemical, Ann Arbor, MI, USA). Following the manufacturer’s instructions, PGE_2_ and PGD_2_ samples were diluted in sample buffer at 1:15 and 1:30 for controls and treatments, respectively. Samples were incubated overnight in the pre-coated plates provided with the specific antibodies and conjugates. Then, a substrate solution was added, and plates were read at 405 nm using a spectrophotometer (SpectraMax). Concentrations in pg/mL were obtained from standard curves, using a four-parameter logistic curve fitting.

### 2.4. Cyclooxygenase-2 Protein Expression by Western Blotting

Cells at 2 × 10^4^ cells per well were seeded in 6-well plates and kept in culture for 48 h. At that time, 10 ng/mL of IL-1β and TNF-α were added in starving media 1% ITS. After 44 h, protein was collected. Cell lysis was performed with Laemmli buffer 1×, using a cell scraper to obtain the protein extracts. Then, 30 μL samples (approximately 20 μg of protein) were resolved by electrophoresis on 10% sodium dodecyl sulphate–polyacrylamide gels under reduced conditions at 90 V for 120 min and then transferred to a nitrocellulose membrane (GE Healthcare, Chicago, IL, USA) using a wet blotter at 90 V for 90 min. The membranes were blocked with 5% non-fat powdered milk and incubated overnight at 4 °C with primary antibody (1:1000) rabbit anti-mouse COX-2 (Cayman Chemical). Then, membranes were washed and incubated with donkey anti-rabbit secondary antibody (1:2000) HRP-conjugated (GE Healthcare). The peroxidase-conjugated antibodies were detected by chemiluminescence (Immobilon Western from Millipore, Billerica, MA, USA). The intensity of the protein bands was quantified by densitometry (Image J, NIH, Bethesda, MD, USA). The constitutive protein β-actin detected by anti-mouse antibody (1:4000) (Sigma-Aldrich) and sheep anti-mouse IgG secondary antibody (1:6000) HRP-conjugated (GE Healthcare) was used as the internal loading control. Results are expressed as the ratio of the relative expression to the control (untreated cells).

### 2.5. Cyclooxygenase-2 Gene Expression by Quantitative PCR (qPCR)

Cells at 2 × 10^4^ cells per well were seeded in 6-well plates in triplicate and kept in culture for 48 h. At that time, IL-1β (10 ng/mL) was added in 1% ITS. After 44 h, total mRNAs were extracted from cultured cells using the RNeasy Mini kit (Qiagen, Hilden, Germany), and mRNA was reverse-transcribed using the QuantiNova Reverse Transcription Kit (Qiagen). qPCR was performed with QuantiNova SYBR Green PCR (Qiagen) and gene-specific primer pairs for *Cox2* (PrimerBank ID 118130137c1) (EXXTEND, São Paulo, Brazil) with sequences described in Table 1. Expression levels of genes of interest were measured using Applied Biosystems 7500 real-time PCR (Thermo Fisher Scientific, Waltham, MA, USA). Amounts of mRNAs were normalised relative to the *GAPDH* gene (PrimerBank ID 6679937a1) (EXXTEND), and the 2^−ΔΔCT^ method described by Livak and Schmittgen was used to calculate the fold change relative to untreated control [40].

### 2.6. Expression of Myogenic Regulatory Factors

Cells at 0.1 × 10^4^ cells per well were seeded in black Advanced TC 96-well microplates (Greiner Bio-One, Kremsmünster, Austria) and kept in culture for 48 h. Cytokines IL-1β, TNF-α, and IL-6 (BioLegend) at 10 ng/mL were added in 1% ITS (for studies in the proliferative stage) or in 2% horse serum (for studies in the commitment stage). After 24 h, cells were washed with PHEM buffer (2 mM HEPES, 10 mM EGTA, 2 mM MgCl_2_, and 60 mM PIPES pH 6.9) and fixed for 1 h with cold 4% paraformaldehyde. Cells were permeabilised with 0.1% Triton X-100 for 5 min and blocked with 1% bovine serum albumin for 30 min. Primary antibodies for Pax7, Myf5, MyoD (Santa Cruz Biotechnology, Dallas, TX, USA), and myogenin (Sigma-Aldrich) were added in 1% bovine serum albumin and incubated overnight at 4 °C. After PHEM–glycine buffer washing (3×), the cells were incubated with a Hoechst DNA-specific stain (1:3000; Thermo Fisher Scientific), and secondary fluorescent antibodies (1:1000; Thermo Fisher Scientific) at room temperature for 1 h, and plates were subjected to high-content imaging analysis (also known as high content screening, HCS) on MetaXpress High-Content Image Acquisition & Analysis Software (Molecular Devices). The system was used to acquire 16 images per well at 20× magnification. Cell quantification based on images were performed according to the following steps. (1) The nuclei was automatically identified within illumination-corrected Hoechst-33342 to definition nuclear boundaries: the intensity above local background was used for finding the nuclei. (2) An internal mask (cytoplasm) was defined by dilating the nuclear mask out to the edge of the Hoechst-33342; the intensity above the local background for the stained area was used to distinguish positive from negative cells. (3) We measured the fluorescence intensity parameters of the transcription factors inside nuclear and cytoplasmic. (4) The quantitative data shown represent the fluorescence intensity of each protein relative to the secondary antibody.

### 2.7. Myokines Release by Multiplex

Myoblast cells at 2 × 10^4^ cells per well were seeded in 6-well plates and kept in culture for 48 h. At that time, IL-1β and TNF-α (10 ng/mL) were added in DMEM-10%. After 44 h, cell-free supernatants were collected and kept at −80 °C until analysed. The MILLIPLEX MAP Mouse Myokine Magnetic Bead Panel (Millipore) was used to detect several myokines in tissue culture supernatants. MILLIPLEX MAP, based on the Luminex xMAP technology, performs immunoassays on the surface of fluorescent-coded magnetic microspheres coated with capture antibodies specific to the mouse myokines IL-6, myostatin, irisin, osteonectin, and IL-15. Following the manufacturer’s protocol, samples were centrifuged at 1600 rpm for 10 min to remove debris. Then, 25 μL of samples, standards, and controls were added to the 96-well plate with 25 μL of the mixed beads previously prepared. The plate was incubated overnight at 4 °C with agitation. Then, well contents were removed, and the plate was washed with 200 μL wash buffer 3 times with the aid of a handheld magnet, and 25 μL of detection antibody was added. The plate was incubated one hour at room temperature with agitation, and 25 μL streptavidin–phycoerythrin was added. The plate was incubated for 30 min at room temperature with agitation, after which well contents were removed. Wells were again washed 3 times, and 150 μL of sheath fluid was added to all wells. The Luminex 200 analyser (Luminex, Austin, TX, USA) was used to acquire and analyse data. Each individual microsphere was identified, and the result of its bioassay was quantified based on fluorescent reporter signals using Luminex xPONENT 4.2 acquisition software.

### 2.8. Statistical Analysis

All experiments were performed at least three times (some of them in duplicate). Data are expressed as mean ± standard error of the mean (SEM). Statistical significance was determined using either one-way analysis of variance (ANOVA) with Dunnett’s or Dunn’s post-test, according to the data distribution, or an unpaired *t*-test for direct comparison, using GraphPad Prism 8.0.2 (GraphPad Software, La Joya, CA, USA). In addition, a principal component analysis (PCA) and partial least squares discriminant analysis (PLS-DA) were performed using MetaboAnalyst version 4.0 (McGill University, Ste Anne de Bellevue, QC, Canada) [41].

## 3. Results

### 3.1. Proliferative Effects of IL-1β and TNF-α Occur through the IL-6 Receptor

**Figure 1:**
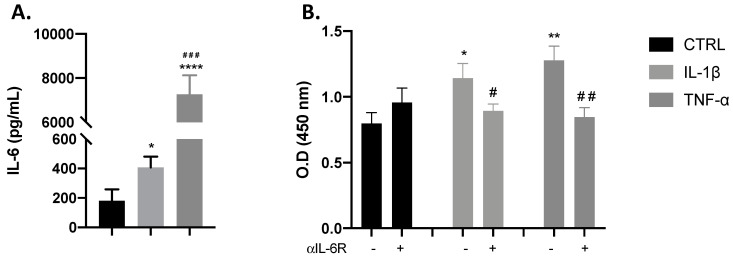
Interleukin (IL)-1β and tumour necrosis factor (TNF)-α effects in proliferation are dependent on IL-6 production. Bar chart shows the effect of control (black), IL-1β (gray), and TNF-α (dark gray) on myoblast IL-6 production and proliferation. (**A**) IL-1β and TNF-α significantly increased IL-6 release. (**B**) IL-1β and TNF-α significantly increased the incorporation of BrdU, which was reduced with IL-6Rα blocking. Data are shown as mean ± SEM (* *p* < 0.05, ** *p* < 0.01, and **** *p* < 0.0001 vs. control from unpaired *t*-test; ^#^ *p* < 0.05, ^##^ *p* < 0.01 and ^###^ *p* < 0.001 vs. IL-1β and TNF-α both from unpaired *t*-test).

As previously demonstrated, the cytokines IL-1β and TNF-α have been described to stimulate myoblast proliferation and to induce the release of IL-6 from these cells [9]. From our results, we confirmed that both IL-1β and TNF-α induced the production of IL-6 (* *p* < 0.05; **** *p* < 0.0001) when compared with control cells, with TNF-α-induced IL-6 significantly greater than IL-1β (Figure 1A). On these bases, we inhibited IL-6Rα to investigate whether IL-6 has a role in myoblast proliferation induced by these cytokines. As shown in Figure 1B, the blocking of IL-6Rα by the pre-incubation of C2C12 cells with anti-IL-6Rα significantly reduced the myotube proliferation induced by IL-1β or TNF-α, as measured by BrdU incorporation. These results indicate that IL-6 is involved in IL-1β and TNF-α stimulation of myoblast proliferation.

### 3.2. COX-2 Pathway Is Involved in IL-1β-Induced Proliferation

#### 3.2.1. IL-1β but Not TNF-α Induced the Release of COX-Derived Mediators

**Figure 2:**
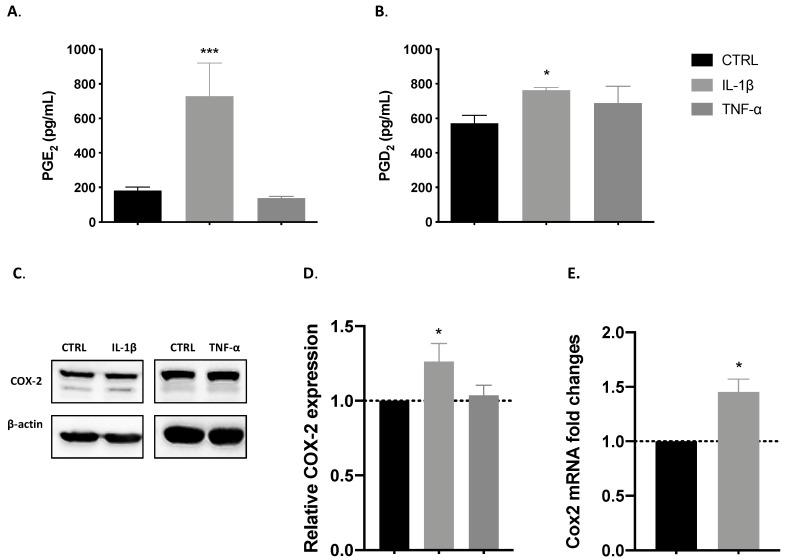
IL-1β induces the release of prostaglandins and cyclooxygenase 2 (COX-2) expression. Bar charts represent effects of control (black), IL-1β (gray), and TNF-α (dark gray) on the release of prostaglandins and on COX-2 expression. (**A**) IL-1β positive effect on prostaglandin E_2_ (PGE_2_) and (**B**) PGD_2_ release. (**C**) Representative blotting of COX-2 protein expression. (**D**) Relative COX-2 protein expression was increased by IL-1β treatment. (**E**) mRNA levels of the *Cox2* gene were also increased by IL-1β. Data are shown as mean ± SEM (* *p* < 0.05 and *** *p* < 0.001 from unpaired *t*-test).

Muscle cells release COX-derived mediators after injury [39], and the activity of the pathway in myoblast proliferation and differentiation is well established [36,42]. As shown in Figure 2A,B, we observed increased PGE_2_ and PGD_2_ release induced by IL-1β (*** *p* < 0.001 and * *p* < 0.5). By contrast, TNF-α incubation had no effect on PGE_2_ or PGD_2_ release. This means that another mechanism could be involved in the TNF-α-induced effect on myoblast proliferation.

#### 3.2.2. IL-1β but Not TNF-α Induces COX-2 Expression

COX-2 pathway activity is involved in muscle regeneration. We investigated the effect of pro-inflammatory cytokines on COX-2 expression at the proliferative stage under starving conditions, evaluating it by Western blotting analysis. The two bands observed in the representative blotting (Figure 2C) are expected for murine COX-2. The presence of a double band is normally detected for these isoforms, because they undergo an *N*-glycosylation in the Asn580 position of the protein structure [43]. Our results demonstrate that IL-1β significantly increased the protein expression of COX-2 in comparison with a control (* *p* < 0.05), as shown in Figure 2D. Working from these results, we evaluated its expression at the mRNA level and found a correlation, with *Cox2* gene expression significantly (* *p* < 0.05) increased by IL-1β treatment (Figure 2E).

#### 3.2.3. IL-1β Induces Proliferation through the Synthesis of COX-2-Derived Mediators

**Figure 3:**
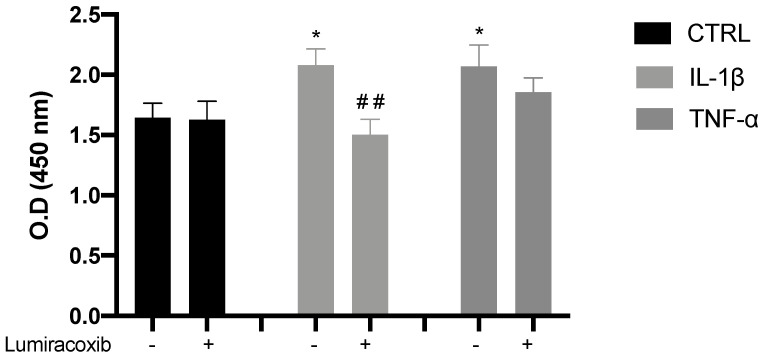
IL-1β effect on myoblast proliferation is mediated by COX-2 activity. Pharmacological inhibition of COX-2 with lumiracoxib leads to a reduction in proliferation, which was increased by IL-1β (gray), in comparison with control cells (black, treated with vehicle 0.1% Tween-80). The TNF-α effect (dark gray) was not affected by lumiracoxib. Data are shown as mean ± SEM (* *p* < 0.05 vs. control; ^##^ *p* < 0.01 vs. IL-1β from unpaired *t*-test).

To determine whether the release of PGE_2_ and PGD_2_ is correlated with the effect of IL-1β on proliferation, we performed a pharmacological inhibition of COX-2, using a recognised tool in the study of muscular regeneration [33,34,42,44]. We found that the positive effect of IL-1β on proliferation was significantly reduced in the presence of the COX-2 inhibitor lumiracoxib (Figure 3). As we expected, no change was found with TNF-α in the presence of lumiracoxib.

### 3.3. IL-1β, TNF-α, and IL-6 Can Modulate the Expression of Myogenic Regulatory Factors in Both Proliferated and Committed Cells Displaying Distinct Effects

**Figure 4:**
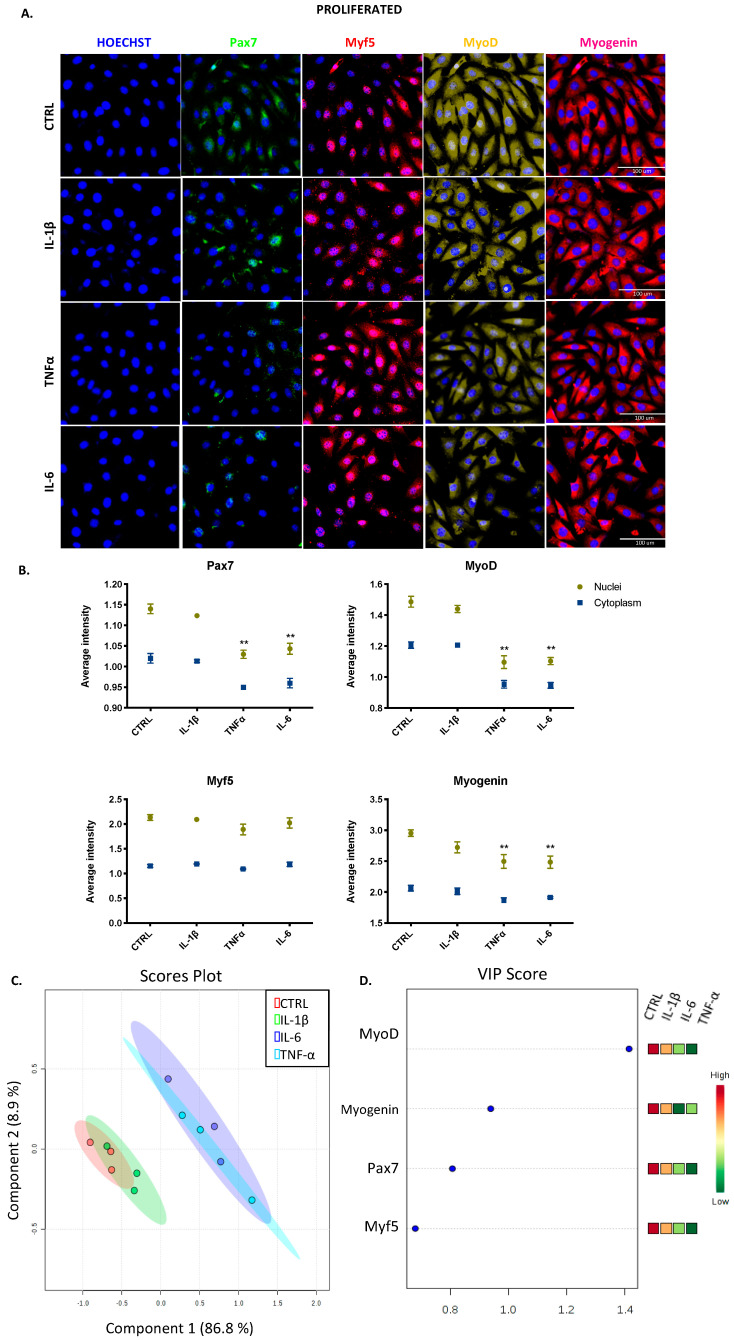
Transcription factor expression for C2C12 cells at the proliferative stage. (**A**) Representative images of myogenic transcription factor expression obtained by high content screening (HCS) (scale bar: 100 μM). (**B**) The average intensity in the nucleus (yellow) and cytoplasm (blue) of every factor at basal (CTRL) and treated conditions. (**C**) PCA and PL-SDA score plots show the differences and similarities between treatments IL-1β (green), IL-6 (blue), and TNF-α (light blue) and the control (red). (**D**) Variable importance in projection (VIP) score indicates that MyoD is the most relevant factor at this stage. Data are shown as mean ± SEM (** *p* < 0.01 vs. control from two-way ANOVA and Dunnett’s post-test; principal component analysis (PCA) and partial least squares discriminant analysis (PSL-DA)).

Since myogenic regulatory factors modulate the progression of proliferating skeletal muscle cells to differentiating cells [5], we used HCS to assess the nuclear and cytoplasmic expression of Pax7, Myf5, MyoD, and myogenin in both proliferation and commitment stages. Any cytokine modified the total cell count (data not shown) at any stage, and basal expression of myogenic regulatory factors (MRFs) was highest in the nucleus than cytoplasm, which means that beyond their expression, we are reporting their activity. Representative images of cells at the proliferative stage are shown in Figure 4A and also in Figure S1, Supplementary Materials. We found that IL-1β did not change the basal pattern of any factor, whereas TNF-α and IL-6 reduced significantly (** *p* < 0.01) the expression of Pax7, MyoD, and myogenin in the nucleus (Figure 4B). The effects of TNF-α and IL-6 were similar, as shown by the overlapped blue and light blue ellipses in the score plots of the PCA and PLS-DA (Figure 4C). The variable importance in projection (VIP) score obtained from PCA revealed MyoD as the most important factor at this stage in our model (Figure 4D).

**Figure 5:**
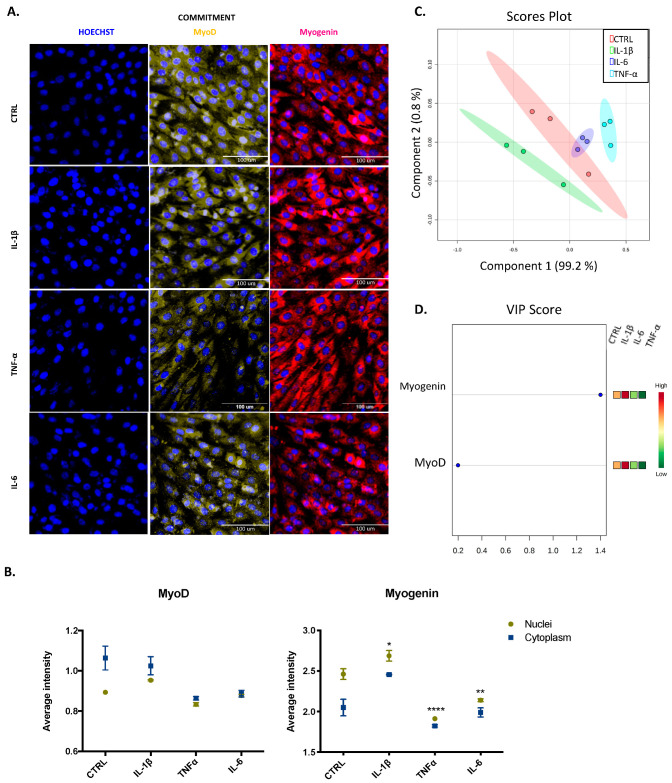
Transcription factor expression for C2C12 cells at the commitment stage. (**A**) Representative images of myogenic transcription factor expression obtained by HCS (scale bar: 100 μM). (**B**) The average intensity in the nucleus (yellow) and cytoplasm (blue) of every factor at basal (CTRL) and treated conditions. (**C**) Principal component analysis (PCA) and partial least squares discriminant analysis (PL-SDA) score plots show the differences and similarities between treatments IL-1β (green), IL-6 (blue), and TNF-α (light blue) and the control (red). (**D**) VIP score indicates myogenin is the most relevant factor at this stage. Data are shown as mean ± SEM (* *p* < 0.05, ** *p* < 0.01, and **** *p* < 0.0001 vs. control from two-way ANOVA and Dunnett’s post-test; PCA and PSL-DA).

For cells progressing to the differentiated stage, representative images shown in Figure 5A and Figure S1 illustrate the commitment phase of C2C12 cells. The expression of MyoD was higher in the cytoplasm than in the nucleus, which reveals its decreased activity. IL-1β increased the activity of myogenin (* *p* < 0.05), but cytokines TNF-α and IL-6 both induced a significant decrease in the expression of myogenin in the nucleus (* *p* < 0.05 and **** *p* < 0.0001; Figure 5B). Each cytokine had a different effect, as shown by the separated green, blue, and light blue ellipses in the score plots of the PCA and PLS-DA (Figure 5C). Myogenin, with high expression in the nucleus, is the most important factor at this stage, according to the VIP score (Figure 5D).

### 3.4. IL-1β and TNF-α Positively Modulate the Production of Myokines

**Figure 6:**
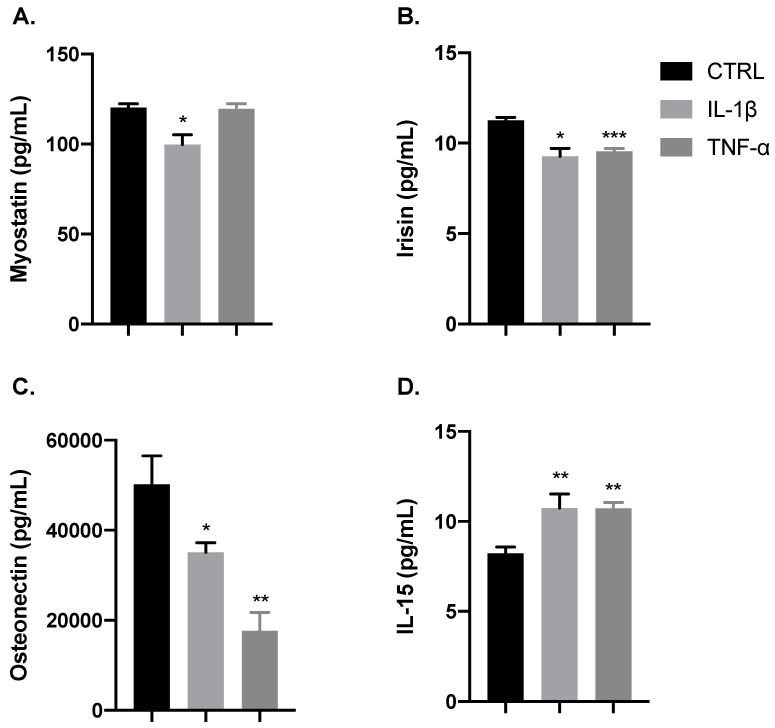
IL-1β and TNF-α modulate myokines release. Bar charts illustrate the release of myokines (**A**) Myostatin, (**B**) Irisin, (**C**) Osteonectin, and (**D**) IL-15. Both IL-1β (gray) and TNF-α (dark gray) reduced the release of osteonectin and irisin, and they increased the release of IL-15 in comparison with control (black). Release of myostatin was not affected by TNF-α. Data are shown as mean ± SEM (* *p* < 0.05, ** *p* < 0.01, *** *p* < 0.001 vs. control from unpaired *t*-test).

Since several myokines are endogenously secreted in response to muscular efforts and are also involved in the growth and repair of damaged myofibers [31], we investigated the effect of the inflammatory cytokines IL-1β and TNF-α on the release of these myokines by myoblast cells at the proliferation stage. As illustrated in Figure 6, both cytokines reduced the release of osteonectin and irisin and increased that of IL-15 (* *p* < 0.05, ** *p* < 0.01, *** *p* < 0.001). In addition, IL-1β but not TNF-α reduced myostatin production (* *p* < 0.05), which is an interesting finding, since it acts as a negative regulator of proliferation.

## 4. Discussion

Skeletal muscle has the intrinsic ability to recover itself after injury, through a complex and highly coordinated process. Muscle regeneration relies on the function of myogenic myoblast cells, and their proliferation and differentiation abilities are regulated by muscular transcription factors, which in turn are critically modulated by inflammatory mediators secreted at the local milieu of muscle repair. Among these mediators, we highlight the pro-inflammatory cytokines TNF-α and IL-1β, which are secreted by immune innate and muscle cells and are involved in inflammatory mechanisms that modulate myogenesis and muscle regeneration after injury [8]. We herein report that TNF-α and IL-1β use similar mechanisms to induce the proliferation of myoblast cells in culture, but they regulate the transitory phase of myoblast differentiation through distinct mechanisms.

Using C2C12 myoblast cells in a proliferation experimental protocol, we observed that both TNF-α and IL-1β directly activate the production of IL-6. These results are in line with previous studies showing IL-1β/IL-6 and TNF-α/IL-6 functional associations regulating the myogenic stimulation of myoblast cells [9,10,45,46]. In our experimental conditions, proliferative myoblasts stimulated by TNF-α secreted higher levels of IL-6 than those stimulated by IL-1β. However, in our experimental conditions, the increase in myoblast proliferation induced by TNF-α or IL-1β did not correlate with levels of secreted IL-6, indicating that myoblast proliferation induced by these cytokines was not dependent on the magnitude of IL-6 secretion. IL-6 is a pivotal mediator of innate response and exerts autocrine effects as a myokine in muscle cells, positively regulating the myogenesis process under repair conditions [23,47,48]. This cytokine exerts its effects by engaging the membrane-bound IL-6 receptor α (IL-6Rα), which binds to the signal-transducing glycoprotein 130 (gp130), triggering signalling mainly by the STAT3 and p38 MAPK pathways, which is known as classical signalling; *trans*-signalling occurs through a soluble form of IL-6Rα and a gp130 subunit [23,48]. Skeletal muscular cells express both IL-6Rα and gp130 [49,50,51]. In this sense, our data demonstrating that the pharmacological inhibition of IL-6Rα reduced myoblast proliferation induced by IL-1β and TNF-α imply that IL-6 has a role in the mechanisms triggered by IL-1β and TNF-α that lead to the proliferation of myoblast cells. Although the ability of IL-1β and TNF-α to increase myoblast proliferation has already been demonstrated [9], to our knowledge, this is the first demonstration of the involvement of IL-6 via IL-6Rα in the proliferation of myoblasts triggered by IL-1β and TNF-α.

Several studies have demonstrated that products from the known induced isoform COX-2 are involved in the modulation of myogenesis processes [42,52,53,54]. Of note, COX-2 expression has been reported as constitutive for C2C12 myoblasts and myotubes [55], supporting its role in muscle physiology. We herein demonstrate that IL-1β, but not TNF-α, has the ability to increase the gene and protein expression of COX-2, leading to biosynthesis of the prostaglandins PGE_2_ and PGD_2_ by C2C12 cells during the proliferative stage. Although our results indicated that TNF-α did not stimulate the COX-2 pathway at this stage, the upregulation of COX-2 expression by TNF-α has been previously demonstrated in C2C12 cells after differentiation in vitro [56]. Studies have demonstrated that prostaglandins modulate proliferation and differentiation events when these mediators are directly added to skeletal muscle cells in culture [36,55,57,58]. The stimulatory effect of PGE_2_ on myoblast proliferation and differentiation has been extensively demonstrated; however, the role of PGD_2_ in these events is still unclear. Only one study has demonstrated that PGD_2_ is capable of inhibiting C2C12 myogenesis, in this case by repressing the mRNA expression of myogenic differentiation markers [57]. Our data demonstrating that proliferation induced by IL-1β can be inhibited by pre-treating myoblasts with lumiracoxib, a selective inhibitor of COX-2, implicate PGE_2_ and probably PGD_2_, produced via the COX-2 pathway, as key mediators of IL-1β-induced myoblast proliferation. This autocrine mechanism triggered by IL-1β in the myogenic proliferative event may take place during the skeletal muscle repair process. Although our data have shown that IL-6 and COX-2-derived prostaglandins are involved in IL-1β-induced stimulation of myoblasts proliferation, crosstalk between IL-6 and COX-2- derived prostaglandins was not presently investigated. Positive feedback loops regulating IL-6 and PGE_2_ production have been reported in different models of inflammation [59,60,61]. Therefore, an investigation of these mechanisms in myoblast proliferation deserves attention.

To better understand the mechanisms by which IL-1β and TNF-α modulate the myogenesis process, we focused on the myogenic transcription factors, which control the synchronised process of skeletal muscle regeneration [5]. The transcription factor Pax7 expressed by quiescent satellite cells induces the expression of genes responsible for proliferation and for commitment to the myogenic lineage. Once activated, these satellite cells also express the proliferative and myogenic markers Myf5 and MyoD, and then progression to commitment and differentiation stages is evidenced by the activation of late markers such as myogenin [5,6,62]. Therefore, MyoD is present in both myoblasts and myotubes, and myogenin is a marker of the transition of C2C12 cells to the differentiation state [6]. In our proliferative experimental conditions, we observed that IL-1β did not change the basal activation of any of the analysed myogenic factors. In contrast, TNF-α and IL-6 decreased the activation of Pax7, MyoD, and myogenin in myoblast cells. These findings agree with previous demonstrations that TNF-α reduced the expression of MyoD and myogenin in myoblasts [26,45], indicating that this pro-inflammatory cytokine is able to break the metabolism of myogenic cells at the initial state of differentiation, delaying the cell cycle exit and thus impairing the progress of muscle repair [17,63,64,65]. Supporting this evidence, our results demonstrated that the activity of myogenin was similarly decreased by TNF-α and IL-6 at the commitment stage, which corresponds to the step before the myoblasts become myotubes. These data are evidence that the downregulation of MyoD and myogenin activation by TNF-α is dependent on mechanisms that involve crosstalk with IL-6. This is in line with previous reports demonstrating that this cytokine and its effector STAT3 are capable of inhibiting MyoD and myogenin expression [26,66,67], leading these studies to associate with the ability of IL-6 to downregulate muscle tissue regeneration in hyperactivated conditions [8,23,47]. Furthermore, evidence indicates that a high local concentration of IL-6 inhibits physiological myogenic progression [8,23,68]. In our study, the IL-6 concentration needed to stimulate myoblast cells in culture was near to that released by TNF-α stimulus. These findings reinforce the idea that the mechanisms involved in the inhibitory activity of TNF-α on muscle tissue differentiation are closely associated with the release of IL-6 and the signalling mechanisms activated by this myokine. In contrast, despite our findings of a direct link between IL-6/IL-6Rα and IL1β-induced proliferation, this cytokine did not affect the transcription factors we studied. This suggests that the high levels of PGE_2_ produced by IL-1β could counterbalance downregulation by IL-6, since PGE_2_ is assigned to accelerate the muscle myogenic differentiation [35,36,58]. Previous reports of PGE_2_-induced upregulation of myogenin gene expression in bovine myoblasts [54] and decreased protein expression of MyoD caused by a non-selective inhibitor of COX in rat soleus muscle support our hypothesis [42].

Regarding the myokines studied herein, our findings that IL-1β, but not TNF-α, decreased the levels of myostatin, a negative regulator of muscle growth and regeneration [69], strongly suggest that this myokine contributes to the effect of IL-1β on myoblast proliferation. Moreover, our data showed that both TNF-α and IL-1β decreased the production of irisin, an important myokine that stimulates myogenesis and muscle growth and is associated with hypertrophy [70,71] and the release of osteonectin, which is a protein associated with the morphogenesis and differentiation of C2C12 myoblast cells [72,73]. These findings lead us to suggest a new mechanism by which TNF-α limits differentiation events and leads to muscle wasting. Consistent with our findings, a previous report demonstrated that TNF-α downregulates osteonectin, which is involved with the differentiation of pulp cells during development and repair [74]. However, the precise mechanisms by which TNF-α and IL-1β downregulate osteonectin and irisin in myoblasts are still unknown and deserve more detailed studies. Finally, our findings that both TNF-α and IL-1β increased the secretion of IL-15, a myokine associated with myogenesis, including autocrine stimulation of mechanisms that lead to hypertrophic effects [75,76], are evidence that this myokine can be an important mediator of myogenesis, one regulated by both TNF-α and IL-1β with a consequent increase of myoblast proliferation.

## 5. Conclusions

In this study, we found that the pro-inflammatory cytokines TNF-α and IL-1β display similar effects at the initial stages of muscle regeneration, but the progression of this process leads to notable differences between IL-1β- and TNF-α-induced effects on maintaining muscle regenerative capacity and avoiding muscle mass loss. First, we showed mechanisms for the positive proliferative effects of IL-1β and TNF-α on myoblast cells through IL-6 via engagement of IL-6Rα, and that the IL-1β effect also involved COX-2-derived PGs. Second, our assessment of the activation of myogenic regulatory factors at the commitment stage evidenced an induction effect for IL-1β but an inhibitory effect for TNF-α for myogenin factor. In addition, both cytokines were capable of modulating the release of most of the myokines studied here. Our results bring new insights into the inflammatory signalling involved in skeletal muscle regeneration, opening new possibilities for exploring the signalling pathways triggered by each cytokine that lead to an increased proliferation of satellite cells and regulate the differentiation process.

## Figures and Tables

**Table 1:** RT-qPCR primers used in this study.

Gen Name	Primer Sequence
*Cox2*	F: TTCCAATCCATGTCAAAACCGT
	R: AGTCCGGGTACAGTCACACTT
*GAPDH*	F: AGGTCGGTGTGAACGGATTTG
	R: TGTAGACCATGTAGTTGAGGTCA

## Supplementary Materials

The following are available online at https://www.mdpi.com/2073-4409/9/9/2005/s1, Figure S1: Representative images of myogenic regulatory factors (MFRs) expression obtained by HCS for C2C12 cells.
