# Supplementary material for: IL-1β and TNF-α Modulation of Proliferated and Committed Myoblasts: IL-6 and COX-2-Derived Prostaglandins as Key Actors in the Mechanisms Involved
Source: Cells. 2020 Sep 1;9(9):2005. doi: 10.3390/cells9092005 (PMC7564831; doi:10.3390/cells9092005)
Supplement: Supplementary file 1 [file cells-09-02005-s001.pdf]

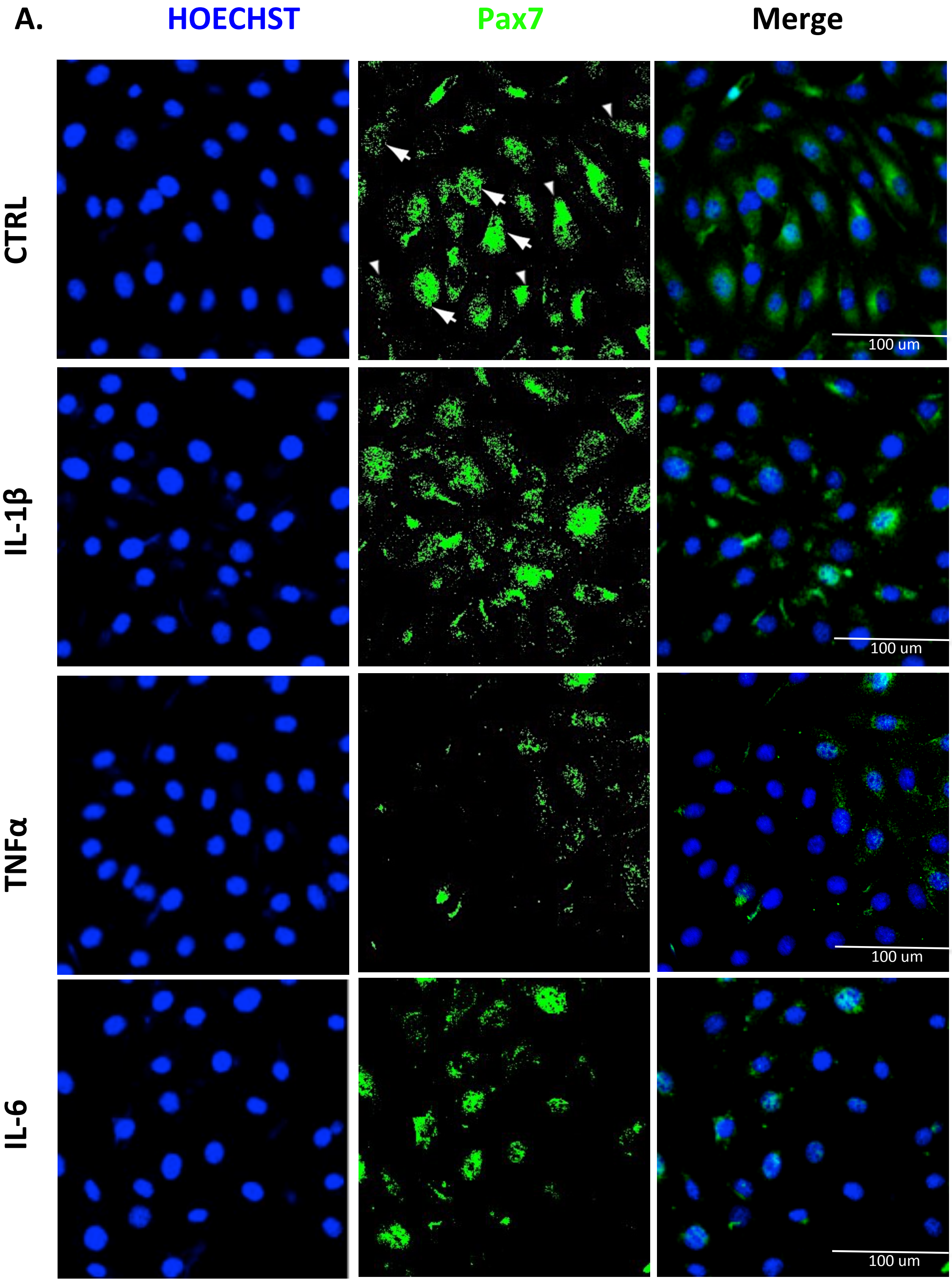
A.
HOECHST
Pax7
Merge
CTRL
IL-1β
TNFα
IL-6
100 um
100 um
100 um
100 um

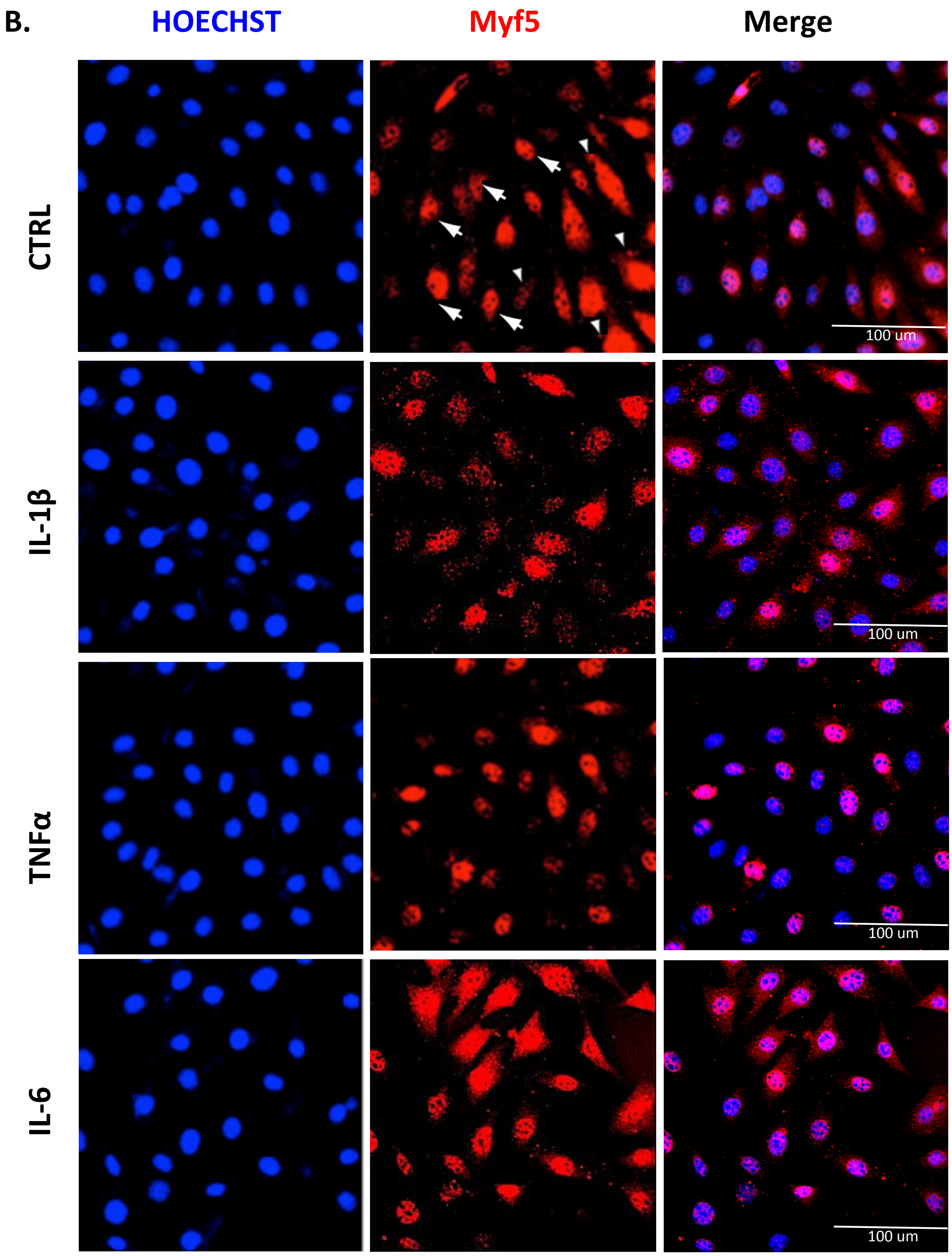
B.
HOECHST
Myf5
Merge
CTRL
IL-1β
TNFα
IL-6
100 um
100 um
100 um
100 um

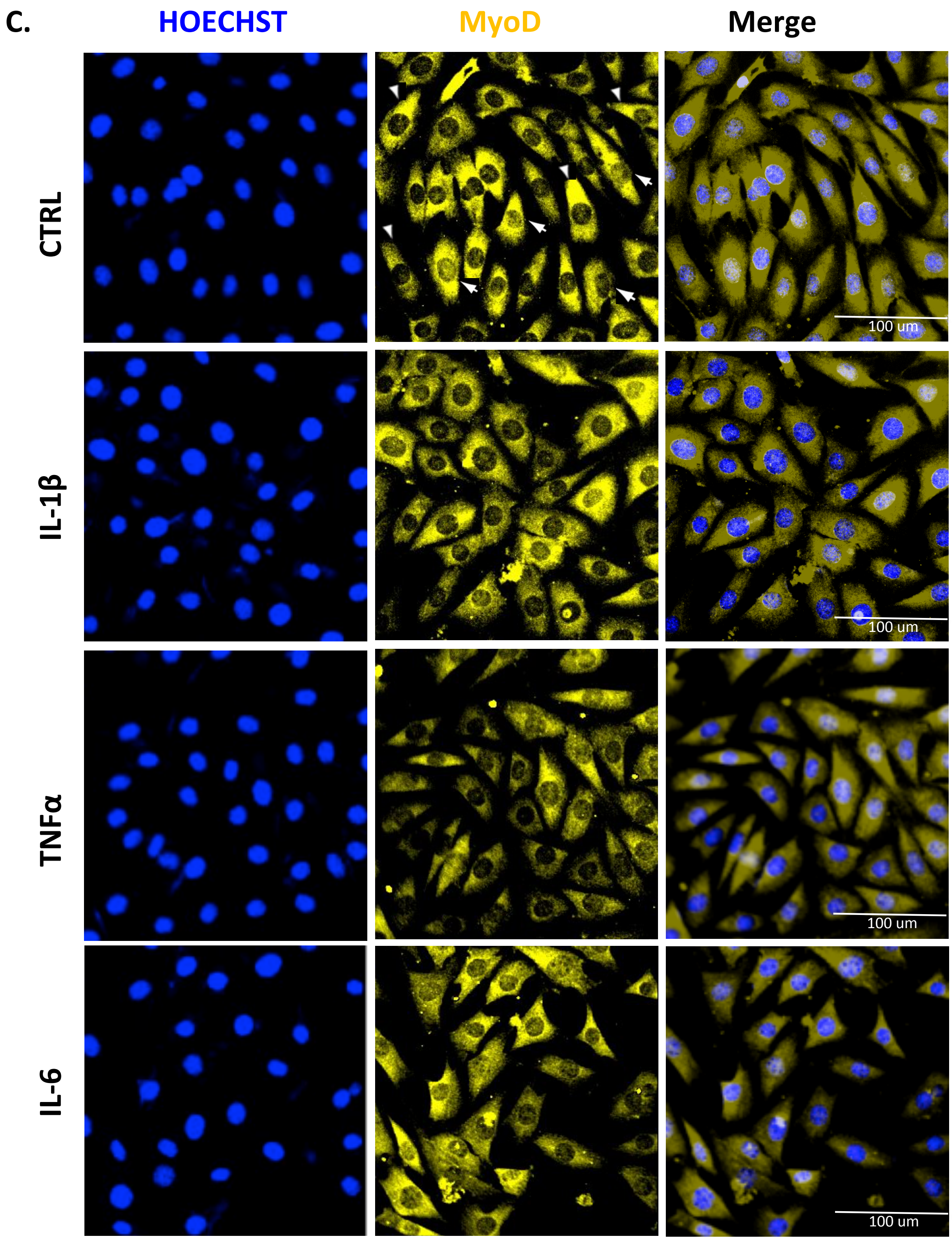
C.
HOECHST
MyoD
Merge
CTRL
IL-1β
TNFα
IL-6
100 um
100 um
100 um
100 um

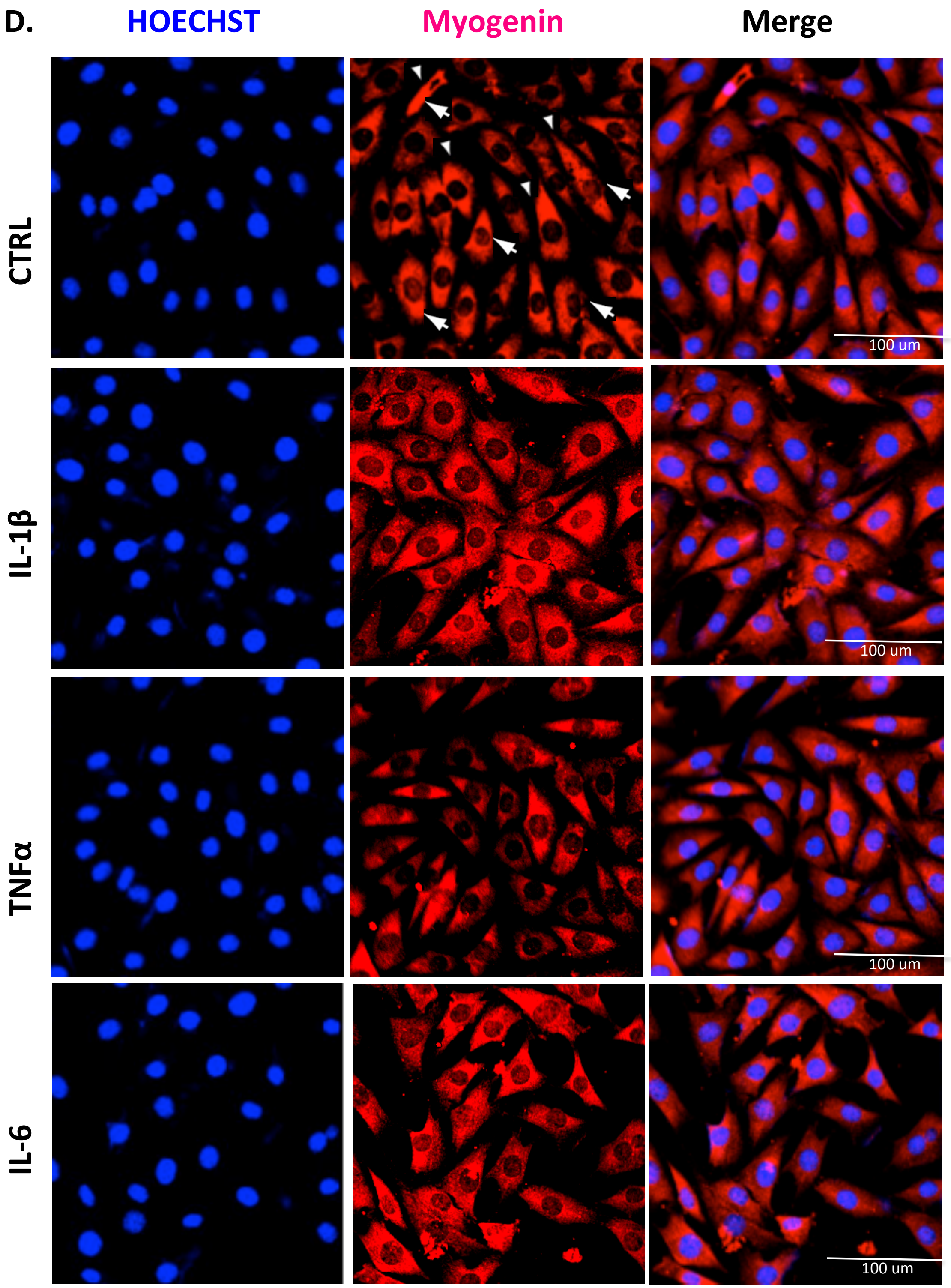
D.
HOECHST
Myogenin
Merge
CTRL
IL-1β
TNFα
IL-6
100 um
100 um
100 um
100 um

E.

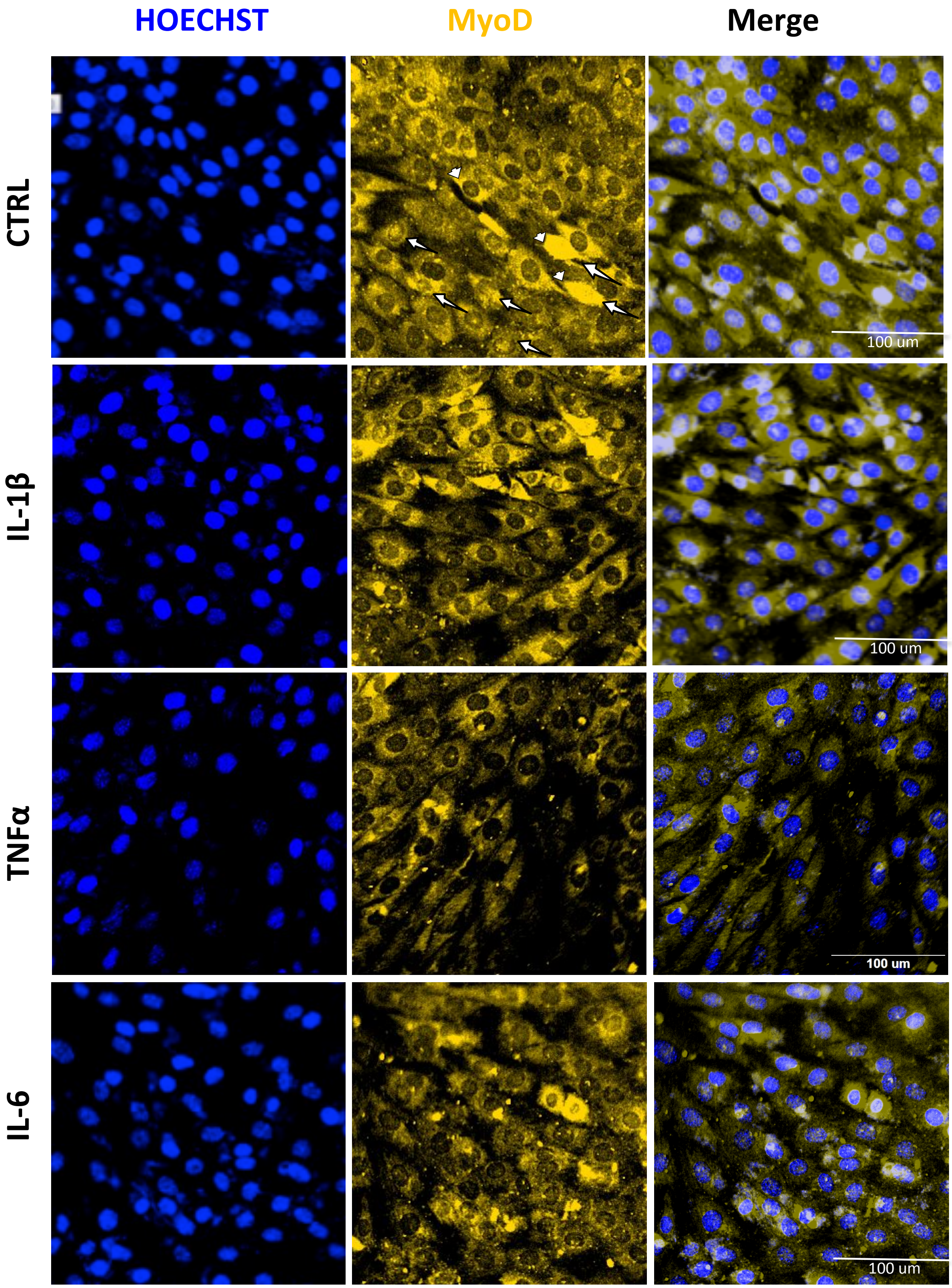

HOECHST
MyoD
Merge
CTRL
IL-1β
TNFα
IL-6
100 um
100 um
100 um
100 um

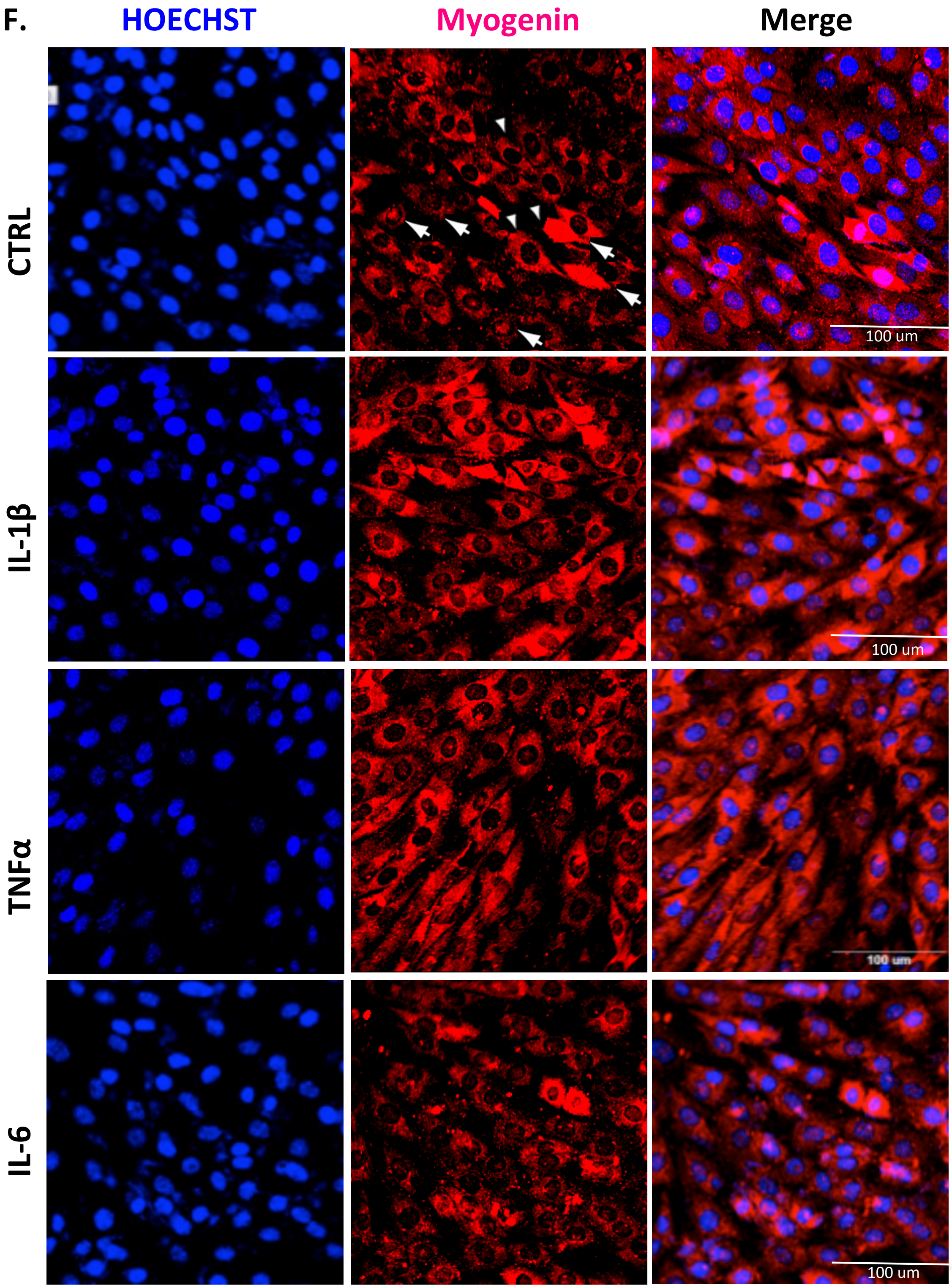
F.
HOECHST
Myogenin
Merge
CTRL
IL-1β
TNFα
IL-6
100 um
100 um
100 um
100 um

Figure S1, **related to Figures 4A and 5A**. Representative images of myogenic regulatory factors (MFRs) expression obtained by HCS for C2C12 cells (scale bar: 100 μm). Non-merged and merged images with Hoechst are presented for (**A**) Pax7, (**B**) Myf5, (**C**) MyoD and (**D**) myogenin at proliferated stage. Images for the commitment stage are presented for (**E**) MyoD and (**F**) myogenin. Examples for nuclear and cytoplasm localization of the MFRs are indicated by arrows and arrow-heads respectively, for the untreated cells (control).
